# Beyond Crop-Raiding: Unravelling the Broader Impacts of Human-Wildlife Conflict on Rural Communities

**DOI:** 10.1007/s00267-024-02018-9

**Published:** 2024-07-19

**Authors:** Wisdom Galley, Brandon P. Anthony

**Affiliations:** 1 Central European University (Department of Environmental Sciences and Policy), Quellenstraße 51, 1100 Wien, Vienna, Austria; 2 Present Address: Dózsa György utca 142, 3572, Sajólád, Hungary

**Keywords:** Human-wildlife conflict, Elephants, Crop-raiding, Kakum Conservation Area, Ghana

## Abstract

This paper examines the impacts of human-wildlife conflict (HWC) in the Kakum Conservation Area (KCA), Ghana. The primary focus is on crop-raiding by elephants. Using ethnographic methodologies, the findings shed light on the broader impacts of HWC in rural communities. These include food insecurity characterized by a notable decline in the quality and quantity of food accessible to individuals and families affected by crop-raiding. The study also underscores the negative impacts on mental and physical wellbeing as residents contend with stress, anxiety and fear due to crop-raiding and encounters with elephants. Furthermore, this research uncovers how coping mechanisms employed by locals in response to these challenges may result in problem drinking. Also, efforts taken to mitigate crop-raiding unintentionally result in health consequences for farmers who face risks of contracting diseases such as malaria and suffer from sleep deprivation due to guarding their fields at night. More importantly, this study provides an in-depth examination of the broader vulnerabilities caused by HWC which are often ignored and underscores the importance of looking beyond the direct impacts in HWC hotspots like KCA.

## Introduction

Typically, human-wildlife conflict (HWC) has been defined as any situation in which human and wildlife resource demands intersect, resulting in the struggle for resources such as food, water, and habitat between humans and wildlife (Madden 2004; Woodroffe et al. 2005; Anthony 2006; Anthony 2007; Anthony et al. 2010). HWC often occurs when wildlife inflict harm on agriculture, livestock, farm-raised game, and fisheries (Peterson et al. 2005). This frequently prompts intentional or retaliatory harm to species prioritized for conservation, instigated by individuals within and beyond the boundaries of protected areas (PAs) (Madden 2004; Lamarque et al. 2009).

Numerous studies highlight the causes of HWC (Madden 2004; Distefano 2005; Lamarque et al. 2009; Lewis et al. 2016; Gemeda and Meles 2018), with increasing human population emerging as a significant factor (Lamarque et al. 2009). The growing competition for the same limited resources and space has compelled the transformation of PAs into ‘islands of habitat surrounded by seas of cultivation and development’ (Madden 2004, p.249). This is particularly prevalent and frequent in rural communities that are in close proximity to PAs (Ogra 2008; Lamarque et al. 2009; Gore and Kahler 2012). The gradual loss and fragmentation of habitat restricts wildlife to smaller pockets of suitable habitat and forces wildlife to stray beyond restricted ranges into adjacent farms and human settlements. The resulting close proximity between residents and wildlife means that conflict in such instances is inevitable.

The direct impacts of HWC, such as injuries, death, crop-raiding and loss of property are well-documented (Madden 2004; Distefano 2005; Woodroffe et al. 2005; Barua et al. 2013; Gemeda and Meles 2018). Human deaths and injuries are the most devastating impact of HWC (Ogra 2008; Lamarque et al. 2009). In some African and Asian countries, mammalian carnivores and herbivores are responsible for numerous fatal attacks and deaths (Binlinla et al. 2014). The consequences of HWC are more severe in developing countries where subsistence agriculture is a significant component of rural livelihoods. These effects are exacerbated by political marginalization, poverty, unresolved compensation payments, and exclusion of local communities in the management process (Ayivor et al. 2013).

In Ghana, the Kakum Conservation Area (KCA) plays a crucial role in creating jobs and generating income for residents through the sale of artifacts to tourists who visit the park and by being employed as tour guides (Acquah et al. 2017). However, it also poses a threat to the livelihoods of the communities around the park (Appiah-Opoku 2011). In 2010, research conducted in KCA revealed that 99% of respondents surveyed agreed that forest elephants (*Loxodonta cyclotis*) were the most problematic wildlife (Addo-Boadu 2010). Dakwa et al. (2016) noted that (i) crops are the basis for range selection by elephants, (ii) cocoa (*Theobroma cacao*), maize (*Zea mays*), cassava (*Manihot esculenta* Cralztz), pawpaw (*Carica papaya*) are commonly raided, and (iii) oil palm (*Elaeis guineensis*) plantations and farms with pepper fence are least affected by raids.

The raiding of farms has a detrimental effect on the community. For example, about 500 households living close to KCA lose approximately 70 percent of their food crops (estimated at 450 USD per farmer) to raiding (Lamarque et al. 2009). In order to manage the situation, residents employ both traditional deterrent methods (burning tires, using obnoxious herbs, noise-making, killing, and trap setting), though these methods are often illegal as they contradict Ghana’s wildlife legislation, specifically Wildlife Conservation Regulations, 1971 L.I. 685 (Addo-Boadu 2010).

HWC also results in the destruction of infrastructure and crop raids. According to Lamarque et al. (2009). crop-raiding is dependent on factors such as the availability of food source, type of food, and the maturation time of crops. For example, Danquah and Oppong (2007) shows that June was the peak month of crop-raiding incursions by elephants along KCA’s border (2.4 raids/km), coinciding with the maturation period of maize, whilst October recorded the least number of crop-raiding activities (0.1 raids/km). On a national scale, crop-raiding by herbivores or carnivores preying on livestock may seem insignificant. However, this can be the difference between self-sufficiency and dire poverty at the household and communal levels.

Conservationists often portray HWC as events that directly impact individuals and groups. Thus, attempts to examine the impacts of HWC tend to gravitate towards the direct or visible impacts whilst the indirect impacts are often not prioritized. However, more recently, some scholars have argued for the need to broaden the impacts of HWC to capture the indirect effects (Ogra 2008; Barua et al. 2013; Khumalo and Yung 2015). In the literature, the indirect effects of HWC are synonymous with ‘secondary impacts’ (Hunter et al. 1990), ‘social complexities’ (Madden 2004), ‘opportunity costs and transaction costs’ (Barua et al. 2013), and ‘hidden costs’ (Bond and Mkutu 2018). According to Ogra (2008), the indirect impacts are characterized by one or more of the following traits: (a) uncompensated, (b) temporally delayed, or (c) psychological or social in nature. Despite their significance, they are often overlooked; hence, they are rarely documented and poorly addressed. Despite the wealth of knowledge on the causes, direct effects and management of HWC in KCA, there is a research gap concerning the broader impacts of HWC.

Moreover, contemporary studies on HWC focus heavily on the direct economic impacts of HWC. For instance, Drake et al. (2021) underscores the inadequacy of economic compensation mechanisms, such as trophy hunting, to offset the substantial losses from crop depredation by elephants. They illustrate that while economic costs are considerable, these do not capture the full spectrum of impacts on affected communities. Kansky and Knight (2014) extend this perspective by emphasizing the importance of intangible costs and negative attitudes towards wildlife, which significantly influence community sentiments and behaviour. These intangible costs are critical yet underrepresented in HWC research, pointing to a gap in understanding how these psychological burdens affect community well-being. Thus, a comprehensive understanding of the broader socio-ecological impacts and the nuanced vulnerabilities experienced by rural communities remains largely unexplored.

Salerno et al. (2020) identify food insecurity as a direct outcome of HWC, particularly from crop depredation by wildlife. They note that while economic and food security impacts are well-documented, the subsequent health implications are not thoroughly examined. The broader vulnerabilities created by HWC extend beyond immediate economic and health impacts. Kansky and Knight (2014) suggest that intangible costs can erode long-term community resilience and social cohesion. These broader socio-ecological impacts are critical areas that require further exploration. Salerno et al. (2021) add that the compounded effects of climate change and HWC exacerbate these vulnerabilities, particularly in terms of food security and community stability. This underscores the need for research that looks beyond direct economic losses to consider the comprehensive impacts on the well-being of individuals.

Our research on the broader impacts of HWC in the KCA addresses this critical knowledge gap and enhances our understanding of HWC in similar contexts worldwide. By focusing on not just the direct economic costs but also the broader consequences of HWC, this study provides a more holistic understanding of how rural communities are affected. The research contributes to a more comprehensive understanding of the broader socio-ecological consequences of HWC and can inform policies that support sustainable coexistence between humans and wildlife.

## Methodology

### Study Area

**Fig. 1:**
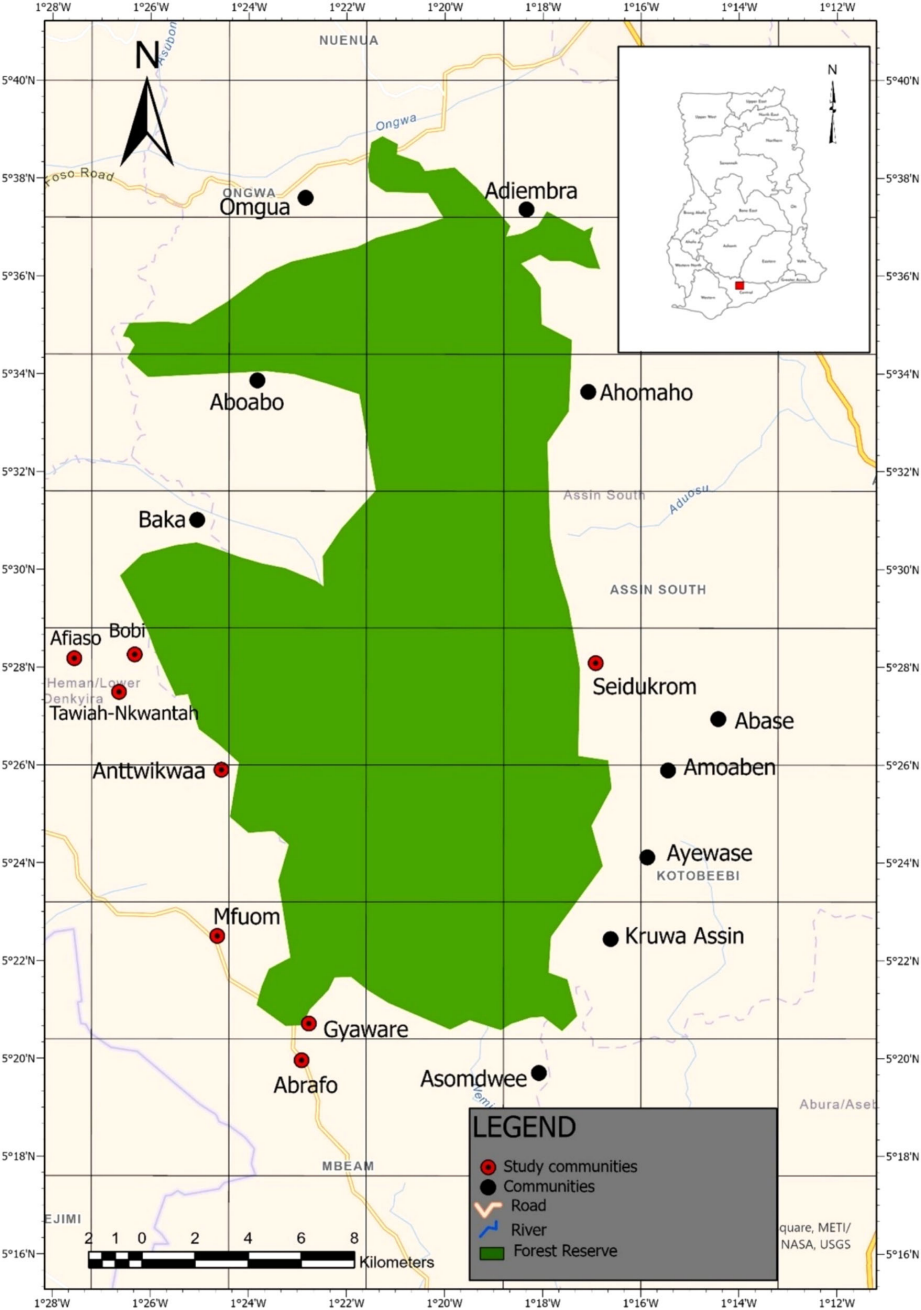
Kakum Conservation Area in Ghana (inset), major surrounding communities (black), including those in this study (red)

The KCA is located 30 km northwest of Cape Coast (Fig. 1) in Ghana’s Central Region (Appiah-Opoku 2011). KCA lies between longitudes 1°30′W–1°51′W and latitudes 5°20′N–5°40′N (Monney et al. 2010), covers an area of 350 km^2^ (Forestry Commission 2024), and comprises two protected areas - the Kakum National Park (KNP) and the Assin Attandanso Resource Reserve (AARR). The dominant vegetation type is tropical evergreen forest (Appiah-Opoku 2011) and is enriched with faunal diversity with about 200 bird species, 600 butterfly species, 200,000 - 350,000 insect species, and about 100 species of mammals (Appiah-Opoku 2011).

Administratively, KCA is under the jurisdiction of the Twifu Hemang Lower Denkyira, Assin (North and South), and Abura–Asebu–Kwamangkese districts of the Central Region of Ghana (Monney et al. 2010). It is amongst the most established ecotourism attractions, with its status as the country’s leading rainforest in this regard (Cobbinah 2015). KCA was declared a forest reserve in the 1930s (Eggert et al. 2003; Fiagbomeh 2012) and subsequently reclassified as a national park and gazetted in 1992 and has since been under the management of the Wildlife Division of the Forestry Commission with support from the Ghana Heritage Conservation Trust.

KCA is bordered by fifty-two communities and more than 400 hamlets. According to Ghana’s 2010 national population and housing census, the villages and hamlets bordering KCA are home to around a quarter of the population of the Assin South and Twifo-Hemang Lower Denkyira districts (i.e., 39, 843 people) (Fiagbomeh 2012). These are primarily rural districts, and the residents are composed of different ethnicities (Twifo, Assin, Denkyera, and Fanti), most of whom are believed to have migrated from the Ashanti Region. In addition to resident ethnicities, other ethnic groups, including Ewes, Krobos, Ga‐Adangbes, and Akuapims, can also be found. These migrant ethnic groups came to settle due to the fertile lands and favourable climate for the cultivation of cocoa and domestic staples (Fiagbomeh 2012).

The adult population of the region has low literacy. Most communities only have elementary schools that last for six years, and none have high schools or tertiary institutions. Over 70% of the residents in districts where the KCA is located are employed in agriculture, while small-scale agriculture employs over 90% of the population in the communities bordering the KCA (Monney et al. 2010). Subsistence agriculture is supplemented by hunting, trading, oil palm processing, charcoal burning, distiling, weaving and domestic animal rearing (Dakwa et al. 2016). Seasonal activities – such as harvesting snails, mushrooms, wood carving and basket weaving – is used by local populations adjacent to the KCA as income-generating activities.

### Data Collection

Taking a social constructionist approach, we utilized local participant observation, interviews, focus groups, and document review for data collection (Burr 1995). The combination of these techniques is advantageous for the broad purposes of depth and the ability to triangulate the data which strengthens the validity of the research (Mertens 2003; Migiro and Magangi 2011).

#### Participant observation

The first author undertook participant observation in selected communities and recorded observations using The Cultural Ecology of Health and Change (CEHC) workbook for descriptive observations of social settings, acts, activities, and events (Whitehead 2005). In the CEHC workbook, observations consist of general descriptions as well as theoretical and reflective notes of what has been observed. A personal fieldwork diary was also maintained during the fieldwork which was a vital component of the data collection process because it supplemented field notes and served as a means of reference.

Field research involved being hosted by key informants in two communities (Abrafo and Antwikwaa). This enabled the familiarization with everyday experiences and also gave a broader perspective of ethnographic hosts’ lived experiences and culture. Also, it enabled blending in through informal conversations, participating in rituals, assisting in agricultural and household labour, attending ceremonies and social gatherings. Staying with ethnographic hosts provided valuable contextual details of the impacts of HWC. Through participant observation, we were able to grasp certain distortions, discern inaccuracies, and verify ‘facts’ obtained through interviews. In addition, it gave access to data on matters that interlocutors were unwilling to share. The use of participant observation as a data collection technique was useful as the goal was to observe the participants in as natural an environment as possible.

#### Interviews

Semi-structured interviews were conducted with key informants, opinion leaders, officials of the Wildlife Division of the Forestry Commission (FC) and individuals affected by HWC. Snowball sampling techniques were used to identify respondents for the interviews. Generally, snowball sampling was utilized to expand and access the social network of respondents. A total of 155 in-depth interviews were conducted in 8 communities: Abrafo, Mfuom, Gyaware, Antwikwaa, Bobi, Afiaso, Tawiah-Nkwantah and Seidukrom. Abrafo was the first community as it is a major settlement and considered the gateway to the park. The other communities were selected after preliminary interviews with park officials and initial assessment of the park’s quarterly reports. The interviews were recorded and later transcribed for thematic analysis.

#### Focus group discussions

Focus group discussions were conducted in Mfuom. The purpose was to gain insights into how both genders are impacted by HWC and understand the range of opinions, inconsistencies, and variations that exist between gender and HWC. According to Colucci (2008), focus groups require consideration of ‘ethnocultural variables’ including group compositions. Hence, in order to diffuse potential power differentials and give space for free expression in a cordial and permissive environment, three focus groups were conducted; (1) men only (6 men), (2) women only (6 women), and (3) mixed (6 men and 6 women). This allowed both genders to express their thoughts freely.

The focus group discussions also afforded the opportunity to observe the nature of communal relations. According to O.Nyumba et al. (2018) the comparisons that participants and interlocutors make of each other’s experiences provide valuable insights into complex power relations and attitudes. In addition, focus group discussions facilitate the clarification and extraction of information from various data sources such as participant observation and interviews.

To supplement the data from the focus groups, interviews and participant observation, a document review was carried out by combing through a total of 30 KCA quarterly reports. Although these park reports were incomplete and poorly preserved, they comprised useful data on the nature of HWC in the communities. Also, reviewing the quarterly reports facilitated the triangulation of data by verifying responses from park officials.

### Data Analysis

Manual thematic analysis was performed to interpret the data. Thematic analysis is “a data reduction and analysis approach in which qualitative data is segmented, classified, summarised, and reconstructed in such a manner that it captures the data set’s key concepts” (Given 2008, p.2). In other words, thematic analysis distinguishes and classifies data according to variations discovered throughout the data collection process. There is no overarching principle or unified approach to conducting thematic analysis. However, it is a method comprised of recognizable measures. It is important to emphasize, however, that this does not imply that thematic analysis proceeds in a static and linear fashion. On the contrary, it is a non-linear and iterative process in which various phases can overlap. The analysis performed is based on six interdependent steps (Table 1).

**Table 1:** Phases of thematic analysis (Braun and Clarke 2006)

Phase	Description of the process
1. Familiarizing yourself with the data	Transcribing data (if necessary), reading and re-reading the data, noting down initial ideas.
2. Generating initial codes	Coding interesting features of the data in a systematic fashion across the entire data set, collating data relevant to each code.
3. Searching for themes	Collating codes into potential themes, gathering all data relevant to each potential theme.
4. Reviewing themes	Checking if the themes work in relation to the coded extracts (Level 1) and the entire data set (Level 2), generating a thematic ‘map’ of the analysis
5. Defining and naming themes	Ongoing analysis to refine the specifics of each theme, and the overall story the analysis tells, generating clear definitions and names for each theme
6. Producing the report	The final opportunity for analysis. Selection of vivid, compelling extract examples, final analysis of selected extracts, relating back of the analysis to the research question and literature, producing a scholarly report of the analysis.

One of the most critical first steps of the data analysis process is tidying up, forming initial impressions and identifying any holes or data chunks by determining if the data collected is enough to answer the research questions. It also entails becoming acquainted with the data. For example, when gathering data, we saw trends, which we noted on a regular basis. While still in the field, we also transcribed and also started reading transcripts of some of the interview data. By doing this, we gained a solid but limited interpretation of the various patterns that emerged from our data set. We coded the entire data collection, resulting in a lengthy list of codes. This means that we removed chunks of text from their original meaning and assigned them a unique code, or identifier that best describes the ‘essence’ of what is occurring in said text chunk. To create themes, we began by checking them for similarities and trends, and then elaborated on the findings and themes in order to give a voice to the ethnographic hosts.

## Study Findings

The findings reveal the myriad impacts, coping mechanisms, and challenges associated with HWC in the context of economic hardship, physical impacts, and mental impacts (Table 2).

**Table 2:** Impacts, coping mechanisms and challenges of HWC

Category	Economic hardship	Physical impacts	Mental impacts
Impacts	- Loss of livelihood affecting income	- Increased workload due to guarding farms	- Stress and anxiety during encounters with wildlife
	- Decreased food availability due to crop-raiding	- Stress and tiredness from implementing deterrent measures	- Fear of wildlife attacks and personal safety concerns
	- Dependency on social networks for food	- Injuries or fatalities from wildlife encounters	- Psychological trauma related to personal safety concerns
	- Alcohol abuse due to coping mechanisms	- Transmission of diseases such as malaria from guarding farms	- Increased risk of mental health disorders due to alcohol abuse
Coping	- Reliance on alternative income sources	- Implementing lethal and non-lethal deterrent measures to protect crops	- Seeking social support networks for emotional relief
Challenges	- Limited economic opportunities and avenues	- Limited resources for implementing mitigation mechanisms	- Social stigma associated with mental health issues
	- Financial burden of repairing damaged property	- Risk of injury or harm while implementing measures	- Lack of access to mental health services in rural areas

### Economic hardship and food insecurity

Crop-raiding by wildlife, especially elephants, via herbivory and stomping poses a danger to the food security in most communities surrounding the park (Table 2). Food security pertains to the consistent access, both physically and economically, to adequate, safe, and nutritious food, meeting dietary needs and preferences for an active, healthy life for all individuals (Kiptot et al. 2014; Agarwal 2018). Additionally, food security entails consuming an adequate number of calories as well as micronutrients to preserve health and nutrition. Thus, food insecurity is the inability to accomplish this goal. Our findings from the focus group discussions and interviews in Mfuom show that crop-raiding by wild animals has a detrimental influence on most households in the community. Although chronic hunger was not a common occurrence in the communities, there was concern about an imbalanced diet as crop loss implies less money to satisfy nutritional requirements. For the past five years, one of the farmers in Mfuom has had her cassava and plantain fields damaged by elephants at least once a year. She stated that the activities of elephants have an impact on the availability of food in her household:

I am a proud farmer. I have always been, but I feel a sense of shame when I have a farm but cannot feed my family. Sometimes I have to borrow money to buy food from other farmers. Is that fair? Toiling and suffering for weeks only for these animals to destroy everything overnight. [Female, 48]

A middle-aged man who endured crop-raiding by elephants in the past year in Tawiah-Nkwanta also expressed his displeasure:

I sometimes rely on other farmers for food. I am not lazy. I am young and work hard, but the destruction of my farm is sickening. They [elephants] worry us. Not just me but my neighbours also. If I get a good job, I will consider quitting because I can’t be a farmer and still struggle to feed myself. [Male, 40]

In the KCA communities, both farmers and wildlife officials identified elephants as the main offenders, similar to other findings (Barnes et al. 2005; Addo-Boadu 2010). Mammals such as wild pigs and elephants are often blamed for agricultural destruction and do bring significant suffering to subsistence farmers (Sitati et al. 2005; Graham et al. 2010; Tiller et al. 2021). However, smaller, more ubiquitous animals are also significant crop raiders that regularly destroyed observed farms, contributing to food loss. These included rats (*Cricetomys emini*), squirrels (*Anomalurus* spp.), grasscutters (*Thryonomys swinderianus*) and birds including red-billed quelea (*Quelea quelea*), all of which affect the food supply of households resulting in decreased nutritional status. Globally, numerous species function as crop raiders, with various degrees of influence depending on the local circumstances. Insects, for example, do significant harm to farms in Africa; birds can also cause significant losses of grain (Bellotti et al. 1994; Stenseth et al. 2003). On the other hand, farmers in our study area did not express much frustration with these smaller species because, comparatively, they caused less damage to crops and were often trapped and used as food, although this is illegal without a permit from the Wildlife Division.

Farmers in the affected communities also reported that crops (such as maize, plantain and cassava) that are critical for the sustenance of their households were the most susceptible to raiding by wildlife. Plantain and cassava are of great economic relevance and sustenance to farmers in the communities. For example, cassava, which is locally known as *bankye* in Ghana, is an important staple crop with per capita consumption of 152.9 kg/year (Adjei-Nsiah and Sakyi-Dawson 2012). In addition to its function as a primary source of nutrition, cassava is a versatile crop that is processed into *gari* (roasted cassava granules), fufu powder, and *kokonte*^1^. In the communities, cassava is grown either as a single crop or as part of an intercropping system with other food crops, aiming to mitigate vulnerability to wildlife raids. It is grown either as the primary or secondary crop; and because of the high nutritional content of cassava leaves, they are used as food for animals. For residents who practice mixed farming in the communities, planting cassava is advantageous as leaves and peels are used for feeding livestock, particularly goats, pigs, and sheep.

Also, according to the farmers, maize was one of the most raided crops. Maize is a favoured food crop in the communities due to its high yield and cheap labour input. It is a significant staple not just in Ghana but in other countries supplying at least 30% of the calories consumed by more than 4.5 billion people in 94 developing countries and more than 20% in regions of Africa and Asia (Shiferaw et al. 2011). Constituting an essential source of food security in the communities in KCA, it is at the same time more sensitive to raiders such as elephants due to its nutritional content (Adeola et al. 2018; Siljander et al. 2020).

Crop-raiding not only reduces the quantity of food available but also limits the variety of food available and thus lowers the quality of food supply. Some farmers during the focus group discussions outlined a connection between nutrition and their health and expressed their displeasure at the presence of elephants as their mobility is limited, making food crops inaccessible. A female farmer remarked:

I am scared. They are huge and fearless. When they are hungry, they are even more courageous. When I see them on my farm, I just go home empty-handed and starve. It is better for me to go hungry for the day than to get injured by these animals. [Female, 50]

The loss of food as a result of HWC is not exclusive to KCA. Studies conducted in other parts of Africa and Asia show crop-raiding by large-bodied animals like elephants is one of the most common forms of HWC, significantly threatening food supply (Ogra 2008; Lamarque et al. 2009; Mayberry et al. 2017; Gemeda and Meles 2018). For example, in Uttarakhand (previously Uttaranchal) near Rajaji National Park in India, 98 percent of survey respondents reported that crop-raiding by elephants had a negative impact on their household’s overall food supply; this notwithstanding, women were found to be disproportionately affected because they are compelled to consume less in order to provide nourishment for their children (Ogra 2008).

Similarly, in other HWC hot spots in Africa, there are similar findings. For example, Nyirenda et al. (2018) demonstrated how crop-raiding elephants in the Lupande Game Management Area impacted the food security of surrounding subsistence farmers in Eastern Zambia. Bukie et al. (2018) observed that crop-raiding affected the food supply of farmers in Nigeria. In summary, crop-raiding by animals has a significant impact on the food baskets of subsistence farmers in terms of food loss, and while the loss of a few hectares of food staples to elephants in a single night may seem insignificant on a national scale, for rural households and families, it may represent the loss of their whole year’s food supply and also be the difference between self-sufficiency and starvation.

This study reveals the profound effects of crop-raiding by wildlife, particularly elephants, on food security and economic stability in the surrounding communities. These findings are consistent with the challenges faced by subsistence farmers around Gishwati Forest in Western Rwanda, highlighting a broader pattern of HWC exacerbating food insecurity and economic hardship in rural areas that are in close proximity to PAs. In KCA, the destruction of important crops such as cassava by elephants leading to a significant reduction in food availability and nutritional quality mirrors the situation in Gishwati Forest, where primates’ preference for raiding maize, a staple food, resulted in substantial nutritional deficits, especially among vulnerable populations (Bush et al. 2010; McGuinness and Taylor 2014). Furthermore, testimonies from farmers in KCA emphasize the economic toll that crop-raiding imposes. Similar economic patterns are evident in Gishwati, where wildlife-induced crop losses can account for 10–15% of household income, highlighting the fragility of subsistence farming (Bush et al. 2010). Given that agriculture is the primary source of income for these communities, any disruption caused by wildlife can push families towards poverty and increased food insecurity. These findings show that addressing HWC requires a multifaceted approach that not only focuses on protecting crops and mitigating wildlife impact but also considers the broader socio-economic context.

The phenomenon of crop-raiding also precipitates financial losses, particularly impacting individuals whose livelihoods hinge entirely upon agricultural earnings (Madden 2004; Distefano 2005; Lamarque et al. 2009). In KCA, we noted that farmers and households possessing diversified income streams are less vulnerable, as they possess alternative revenue sources. The depletion of agricultural fields due to crop-raiding not only introduces economic instability but also jeopardizes potential safety nets during periods of adversity. Furthermore, farmers also conveyed a loss of personal dignity and identity when confronted with the aftermath of crop-raiding, further exacerbated by their reliance on external assistance especially in instances where crop-raiding renders them wholly reliant on the goodwill of their kin and acquaintances.

The shame that most interviewed farmers feel about the inability to sometimes be food secure extends beyond their families. The pride they associate with their profession can be sensed in the sceptical way some farmers discussed the idea of compensation as a strategy to manage the conflict.

“There is no dignity in seeing our children go hungry whilst we pride ourselves of being farmers’ [Male, 45]

Another farmer retorted: ‘*We have been having losses for almost every year. It will be a nice gesture if the government can compensate us for the losses. However, we don’t want to be seen as beggars, we are proud farmers, and we just want the right thing to be done’* [Male, 39]

The complexity of his emotions is illustrative of the complex nature of pride in general among farmers. He is relieved that he will be able to get some compensation for his losses but he is disappointed that he will also lose his *raison d’être* because the farm is an integral element of the farmer’s identity and sense of pride (Brandth 2002; Burton 2004; Heather et al. 2005). For most of the farmers, farming is not just another job. The feeling of belonging to a bigger unit, one that extends beyond the boundaries of one’s own farm and one’s own generation, is encapsulated in being part of a unique heritage.

‘We will always be farmers; this is what we are good at. My sons are in high school, but they are skilled farmers too, I am sure one of them will take over my farms when I am gone’. [Male 48]

‘Farming is what I have known all my life. It is not the most lucrative job, but I am proud of what I do. I am sure my family will still continue when I am old and weak.’ [Male, 55]

Some of them also take pride in the fact that their farms are a source of employment for many people.

I am a farmer; my father was also a farmer and so too was my grandfather. Farming is what we have always relied on to make ends meet. The whole family invests a lot of time and money into farming. Unfortunately, in recent times, our efforts are in vain because the animals eat and destroy everything. [Male, 54]

‘These staples are needed wherever you go. I am proud to be a farmer’. [Male, 50]

‘The cassava and plantain we farm here are sometimes sold in the city and consumed by a lot of people who have never been on a farm, and have no idea what it takes to produce these staples’ [Male, 45]

The revenue most farmers earn from selling their produce is often used to purchase services and commodities. As a consequence of the loss of income, they lose access to necessities, and their quality of life declines.

The money I get from selling maize and cassava is what we all rely on. Everything we buy is from money from the farming business, so it is difficult when the farm is raided by elephants [Male, 45]

Also, income gained from farming is often used to cover a range of household expenses such as children’s tuition, books, uniforms, rent, and medical care amongst others. When crop-raiding deprives a farmer of economic stability, the livelihood of the whole family is impacted, including the aged. Also, in such circumstances, farmers are sometimes unable to buy materials needed to protect their crops. Thus, when a farm is raided, farmers lose both the money required to survive in the present and the income needed to diversify their portfolios for the future.

### Physical Health Impacts

HWC has a direct physical impact on humans, as interactions with wild animals can be risky. In many rural communities situated near PAs, deaths and personal injuries resulting from wildlife attacks are common (Ogra 2008; Lamarque et al. 2009; Binlinla et al. 2014). For example, in India, about 400 people are killed by elephants every year (Rangarajan et al. 2010), and in 2005, 157 crocodile attacks were recorded in the Caprivi region in Namibia (Lamarque et al. 2009). In Nepal, across Terai and Chure regions, for the period 2000–2020, there were 412 reports of elephant attacks on people (274 deaths and 138 injuries). Elephant attack victims varied in age from 7 months to 80 years, although the majority (71 percent) were adults between the ages of 25 and 64 (Ram et al. 2021).

However, during our research in the communities, we found no evidence of HWC-related injuries or deaths in the park archives. The interviewed participants, key informants, and park officials were also unaware of any HWC-related injuries or deaths in the communities. In the mixed focus group discussions in Mfuom, participants attributed the absence of injuries and casualties to their knowledge of animal behaviour passed on to them by their parents:

In my eyes, they are humans because they are protective of their babies, so I always keep my distance when I see them with their babies, and they hardly come to the farm alone. They always come around with their herd, and that makes them braver. Whenever I see them, I am extra cautious and walk back home. [Male, 46]

These creatures are bigger and taller than me and they are very intelligent animals. When I meet them on my farm. I stand at a safe distance and try to scare them away. They don’t mind because they know I am scared, and I will not harm them. So, I usually call the wildlife officials, but most often nothing is done. [Female, 50]

Despite their rich knowledge of wildlife behaviour and the absence of casualties, participants still expressed great worry and concern about the possibility of injuries and casualties from wildlife attacks in the future. They expressed their everyday dissatisfaction and fear. Thus, even the fear of elephant crop-raiding generates distress and apprehension, regardless of whether an elephant raids their crops or not:

Elephants are big. They can easily kill a human being. I have been farming in this community for many years and I have not heard that an elephant has killed anyone [Female, 42]

Moreover, despite the absence of casualties, participants still expressed concern about stress and sleeplessness and the impact on their general wellbeing. Although stress and sleeplessness are a ‘hidden’ cost of HWC since it does not involve direct human-wildlife contact, as with mental health issues, physical injury and stress may all affect an individual’s capacity to work, hence exacerbating their already poor circumstances. It was observed that farmers in the communities regularly engaged in various labour such as tilling, weed and pest control, harvesting and selling their produce on the local market or to middlemen/women; and the lack of sleep due to guarding fields from their huts at night causes them significant stress. It was also observed that most of the farmers were exhausted and had a variety of symptoms, including weariness and bodily pains. They expressed concern about their lack of sleep and fatigue:

I don’t get much sleep in the harvest season. I guard my farm day and night. I stay on the farm and do my best to drive them away when I see them. If I don’t do that my family and I will starve. It is better for me to stay up in the night than go hungry. [Male, 50]

Crop-raiding is a well-known experience amongst local farmers, many of whom are proud, devoted, and industrious. For example, an old man from Mfuom described how the elephants occasionally destroyed his crops, almost leaving him with nothing. He said his pepper fence and intimidation tactics only temporarily protected the crops. *‘The elephants keep coming back almost every year*,’ he added, pointing to the direction they usually come from. *‘They ruin everything*.’ He added that other animals were also guilty. ‘*Not just elephants, birds, and even grasscutters sometimes eat the crops*’, he remarked.

He described how he keeps an eye out for any signs of elephant activity day and night, and makes noise or uses a torch to scare them away. *‘This is my second home*,’ he said, indicating the hut. *‘I made it myself*.’

After the interview, he showed his neighbour’s property, which had similar issues. He emphasised that the hut is effective in helping to fight human thievery and wildlife raiding. Despite its humble appearance, it reflected the resilience of farmers.

Like other farmers spoken to, he recalled the gruelling hours he spent in the fields to guard his farm. He spoke about the animals that had destroyed his crops. ‘*I work hard daily to secure these crops*,’ he yelled. *‘Every year, the elephants destroy everything*.’ Our conversation revealed how widespread the problem of HWC is in the communities. He also explained how he and other farmers had experimented with various crop protection techniques. Yet, the wildlife could get through, resulting in significant crop losses.

While we talked about the animals and crops, the conversation moved along swiftly and intermittently light-hearted. Nevertheless, his wife spoke up. She exclaimed, *‘we are getting tired of talking. We want some action!*’, referring to the inability of wildlife officials to find a solution to the problem. The loss of their crops was much more than a minor inconvenience threatening their hard work and dedication. It was a fear that terrified their community’s way of life and most of the farmers were eager to make their sentiments known.

As old as I am, I do everything myself. I plant, guard, harvest and sell the produce by myself. I spend the little money I get on food and medicine for my pain. Sometimes I feel like giving up but if I stop farming what will I eat? [Female, 52]

Farmers often try different deterrent strategies. One of the most popular measures is the use of chili pepper fences. However, we discovered that pepper fences did not prevent significant crop loss in the communities, even when combined with other deterrent tactics. On many occasions, the fences were flawed. Elephant(s) evaded these fences and continued to damage farms. This is unsurprising, considering elephants’ inclination to navigate around obstacles when presented with an opportunity to do so (Thouless and Sakwa 1995; Vezina et al. 2019). For other farmers, chilli fences have been effective in deterring elephants. They use string barriers, often with many lines, fastened to tee-shirts laced with chilli and grease or engine oil. Although this strategy is popular amongst farmers, it is expensive for poorer families and can be very labour intensive during the rainy season.

The concerns of farmers regarding their physical well-being are worth noting because sleep deprivation and exhaustion are significant impacts of HWC, with possible consequences for morbidity (Pilcher and Huffcutt 1996; Harrison and Horne 2000; Orzeł-Gryglewska 2010). The medical literature shows that sleep deprivation results in physiological stress, which has a detrimental effect on the immune system (Meerlo et al. 2008). Also, higher stress and inadequate sleep may increase the risk of cardiovascular illnesses and death (Meerlo et al. 2008). Ultimately, when farmers suffer from more significant chronic sickness and have weakened immune systems, their capacity to farm or engage in income-generating activities is limited, thereby putting more stress on the entire household.

Furthermore, farmers in the study area said they utilized a variety of conventional methods to minimize or control crop loss. These include guarding, pepper fences, fires, burning car tires, and the use of scarecrows amongst others. Guarding was the most prevalent approach utilized. It was effective in protecting food staples such as maize throughout its developmental stages. This finding is similar to those in Zimbabwe and Ghana (Gandiwa et al. 2012), where guarding was ranked first in protecting crops during the farming season. Although guarding was a principal approach for preventing damage to crops, it is a time-consuming task. During the mixed focus group discussion in Mfuom, some farmers said protection was provided all year for farms but intensified when the crops start flowering until they are harvested.

On average, during peak crop-raiding months, men spent three times as much time (5–6 days) protecting their farms every week than they did at other times of the year. Crop guarding was mostly done by men. In all eight communities, some interviewees admitted that some of their children under the age of 18 also helped in guarding farms. Some participants indicated their children mostly guarded on school vacations, public holidays, and weekends. In Uganda, similar research revealed two-thirds of all farm guarding activities were undertaken by women and youngsters between the ages of six and twelve (Hill 2000). According to participants of our focus group discussions, in their bid to protect their fields against crop loss, they become vulnerable to mosquito-borne illnesses like malaria. This is not unexpected. Indeed, studies conducted in India show an overlap between malaria-infested areas and human-elephant conflict (Dhingra et al. 2010). Participants of the focus group discussions emphasized the connection between guarding and exposure to mosquitoes. A woman during focus group discussions put it this way:

No one is exempt. We all get malaria; it is not a good feeling, but we are used to it. The mosquitoes are everywhere, both on the farm and at home. [Female, 40]

Men also expressed similar concerns:

There are lots of mosquitoes and other insects on the farm. Staying on the farm to guard, especially in the evening and at night exposes us to lots of bites from mosquitoes. [Male, 60]

Malaria has a significant financial impact on people and families. When individuals are bitten by the *Anopheles* mosquito and infected with *Plasmodium falciparum*, they become ill. This takes the shape of physical pain, emotional distress, and lost leisure time owing to sickness. It has a detrimental effect on one’s health and finances, resulting in destitution. Malaria imposes a greater burden on poorer and more vulnerable families, particularly when the ill person is the breadwinner of the family. Additional household duties may be diverted away from economic activities in order to care for sick relatives. Also, time off work results in a decrease in family income, further impoverishing poor families.

The process of getting therapy imposes a financial burden on families in the form of treatment and prevention expenditures (Asenso-Okyere and Dzator 1997; Patouillard et al. 2017). During the illness, the person may be unable to work totally or may work partly owing to the disease’s debility on a temporary basis. Such circumstances might have a detrimental effect on household productivity (Asiamah Maxwell et al. 2014). Foregone income through opportunity costs associated with contracting malaria and seeking treatment contributes to poverty at the household level. Thus, time and money in seeking treatment or purchasing medications is a significant indirect cost component (McElroy et al. 2009; Ibrahim et al. 2017).

### Mental Health Impacts

When it came to mental health stressors, most participants said the activities of elephants had the most impact on their mental health, citing chronic fear and worry. According to farmers, elephants and the destruction they caused to their farms made them uneasy, which had a detrimental influence on their mental health. These fears and anxiety stem from the possibility of injuries or death from physical attacks from elephants. These fears are understandable as human casualties are the most severe manifestation of HWC. Although the raiding of farms and loss of crops through raiding may be accepted, the loss of human life might elevate the conflict to a new level. Even a single death may worsen the already fragile animosity between the locals and the park officials, and retaliatory wildlife killings may escalate, undermining the primary aim of the park – which is to conserve biodiversity.

These animals are unpredictable, and I fear them. I am not ashamed to admit it. They are scary. I fear they might attack me or even my neighbours. [Male, 40]

Last year, I was nearly injured by an elephant on my farm. I was trying to chase them away but one of them charged at me. I got scared and ran. Every time I go to my farm to work. I still fear a little. [Male, 45]

There is also anxiety and fear of losing large parts of the proceeds of their farms because of the amount of damage the animals can cause.

My fear is that I might wake up and all my crops will be destroyed by elephants. I try to guard my farm as frequently as I can. On days that I am not able to guard I still can’t sleep because I am always thinking if the elephants are on my farm or not. [Male, 45]

In addition to concerns about food security and the risk of injury and death, participants also complained about stress and tiredness. According to the farmers, they put in much work both day and night in providing diligent field security to dissuade raiding elephants. Consequently, the lack of sleep caused by securing fields at night causes extra stress.

I have no idea when they will come. Sometimes they come to the farm when I am around, and sometimes, they come when I leave. I do not get any sleep, especially when my crops begin to mature. [Male, 45]

Others made the connection between lack of sleep and their physical health. Here is how one farmer expressed it:

I am aging faster; I can feel it because I do not rest or sleep enough. I wish I could get enough sleep and just enjoy life, but I cannot. There is no doubt that I will be happier and healthier if I get enough rest. [Female, 55]

The fact that she is impoverished severely limited her capacity to cope. Many others are in a similar position. In KCA, many interviewees in Abrafo voiced persistent anxieties or concerns, suggesting elevated stress levels and poor mental health conditions. These persistent fears and concerns were specifically linked to possible physical health consequences such as injuries and the possibility of casualties in the future. Elsewhere, the mental health impacts discovered can be more severe. For instance, Chowdhury et al. (2008) reported that around half of the women who lost spouses to tiger and crocodile attacks in India’s Sundarban experienced psychological issues as a result of their inability to reclaim their loved ones’ remains for proper funerals. Numerous individuals had significant rates of suicide ideation and depression. Jadhav and Barua (2012) revealed that elephant-related injuries, fatalities, or physical threats exacerbated pre-existing medical disorders and contributed to the development of new ones including post-traumatic stress disorder. The mental health impacts can extend to the families of direct victims as families may experience worry, despair, or other psychological impacts if an elephant attack disables or kills the family’s primary breadwinner or other significant person or persons (Ogra 2008; Barua et al. 2013).

Furthermore, crop-raiding by elephants has a cascading effect, amplifying existing problems such as problem drinking. Participants in the all-male focus group in Mfuom made the connection between HWC and the abuse of alcohol. We also observed that farmers, both young and old regularly abused ‘*akpeteshie*,’ – a popular home-brewed alcoholic spirit made from palm wine or sugar cane juice.

Culturally, in the communities surrounding the park, men are expected to be the breadwinners of the family, and the inability to do so comes with a certain stigma. As a result, some of them use alcohol as a coping mechanism to deal with the discomfort associated with the loss of their livelihood by regularly visiting pubs or beer bars as they are called locally. The participants claimed that drinking *akpeteshie* gave them the confidence to drive away elephants from their farms, a perilous task that requires substantial risk. One farmer explained it this way:

When I drink, I can say a lot of things on my mind. I am not a violent person, but I can be aggressive. I feel the officials don’t see how serious most of us are about this issue. I drink and go and complain to them. I say a lot of things. Sometimes I insult them, and I can only do so when I drink. [Male, 55]

We surmise that the heightened consumption of *apeteshie* while guarding fields poses a considerable risk, potentially increasing the likelihood of casualties and injuries related to encounters with elephants. *Apeteshie* typically contains an alcohol volume ranging from 40 to 50% (Akyeampong 1996), and excessive consumption can profoundly affect sensory perception, leading to behavioural disturbances such as diminished self-control, awareness, and impaired judgment. These impairments during field guarding could potentially result in fatal consequences.

The significance of alcohol as a ‘coping mechanism’ in HWC-related risks is important but not unexpected because alcohol misuse is highlighted from a social learning viewpoint as a regular maladaptive coping response (Abrams and Niaura 1987; Maisto et al. 1999). People who have limited coping options are more prone to drink in response to stressful events or conditions in their daily life. Thus, dependence on increased alcohol use to alleviate distress has been linked to a decreased chance of sobriety (Smith et al. 1993). These observations corroborate Maisto et al. (1999), who found that when an individual’s alternate coping mechanisms are limited, alcohol usage rises in stressful conditions. The hazard associated with this form of drinking is that it jeopardizes family bonds and marital relationships. This was emphasized by many female participants, who noted that although drinking *akpeteshie* may provide their partners with temporary comfort, it merely helps them become negligent spouses.

Although some participants openly acknowledge their alcohol abuse, attribute it directly to HWC, and identify it as a significant coping mechanism amidst the challenges posed by HWC, one cannot overlook the influence of cultural norms and traditions on alcohol consumption within the communities. Even without the presence of HWC, a baseline level of alcohol consumption may persist due to entrenched drinking cultures. In the communities, alcohol consumption is deeply ingrained in social gatherings, religious ceremonies, and daily routines (Akyeampong 1996; Yawson et al. 2015; Hormenu et al. 2018). This cultural persistence underscores the complexity of understanding and addressing the dynamics of HWC, as it necessitates a nuanced understanding of the community’s social fabric. Nevertheless, it would be remiss to disregard the significant role of alcohol as a maladaptive coping mechanism specifically in response to HWC in KCA. This context-specific stressor adds another layer of complexity to the issue, necessitating targeted interventions that address the unique challenges posed by HWC. Consequently, these evidently show that interventions for HWC-affected communities require a multifaceted approach that considers both the broader societal factors and the unique challenges posed by wildlife conflict (Peterson et al. 2010). Thus, by understanding the interplay between socio-economic, cultural, environmental, and individual factors, stakeholders can develop targeted interventions that are ‘socially just, and potentially more effective’ (Ogra 2008, p.1420).

## Discussion

### Poverty and HWC Interplay

Agriculture is crucial for achieving food security, particularly in rural communities in Ghana (Bawa 2019). However, the prevalence of HWC poses significant challenges to this goal. In this study, all participants reported negative impacts from HWC, specifically through crop-raiding. While these losses might seem minor on a national scale, they impose a substantial burden on subsistence farmers, who are often among the world’s most disadvantaged populations (Distefano 2005; Lamarque et al. 2009). In KCA, the most prevalent form of HWC is crop-raiding by elephants, leading to considerable financial damage for farmers who rely on agriculture for their livelihoods. These challenges exacerbate food insecurity, particularly for those already living in poverty (Amoah and Wiafe 2012).

The Central Region of Ghana (where KCA is located), like many other regions, faces a high poverty rate. Nearly 47% of its 3 million residents live in poverty, making it the sixth poorest of the country’s traditional regions (UNDP 2020). This widespread poverty affects various sectors, including the dynamics of HWC. The intricate nexus between poverty and HWC hinders mitigation efforts (Amoah and Wiafe 2012; Binlinla et al. 2014). A key factor linking poverty to HWC is the limited availability of alternative livelihood options. In KCA, the affected communities often have fewer income-generating opportunities, increasing their vulnerability to the economic repercussions of HWC. Poverty also compels communities to intensify agricultural activities to meet their needs, often leading to encroachment into wildlife habitats. This escalation of HWC not only undermines human livelihoods but also threatens wildlife populations. Thus, retaliatory behaviours and criminal activities are further consequences of poverty, as impoverished communities often resort to poisoning, hunting and poaching to recoup their losses (Binlinla et al. 2014). These actions aggravate HWC, damage ecosystems, and compromise conservation efforts. This situation is not unique to HWC-plagued communities but is prevalent in many rural areas, especially where communities heavily rely on natural resources for their sustenance and needs (Lamarque et al. 2009).

For many rural communities, adjacent parks offer critical resources, including timber, non-timber forest products, and various ecosystem services. However, overexploitation, illegal logging, and unsustainable forestry practices have resulted in deforestation, habitat loss, and ecological imbalances. Such overreliance on natural resources can lead to their depletion, intensifying competition between humans and wildlife. In KCA, for example, communities continue to expand agricultural activities and exploit forest resources to meet their needs, causing habitat loss, fragmentation, and degradation (Addo-Boadu 2010; Amoah and Wiafe 2012). This encroachment increases the proximity between humans and wildlife, thereby heightening the likelihood of conflicts and exacerbating their impacts. Furthermore, this dependency on natural resources impedes livelihood diversification, rendering these communities more susceptible to economic shocks and limiting their capacity to adapt to changing conditions (Fiagbomeh 2012). The economic challenges faced by these communities are exacerbated by the lack of compensation, restricted economic opportunities, and inadequate mechanisms for revenue sharing.

According to the Ghana Revenue Sharing Action Plan, the Ghanaian government currently does not allow revenue sharing from national parks, directing all Department of Wildlife revenues to Ghana’s Consolidated Fund (USAID 2000). Presently, entrance fees from six national parks in Ghana are collected and sent to the government Consolidated Fund. The exception is KCA, which has a revenue-sharing system due to its unique canopy walkway. A negotiated portion of the entrance fee, primarily attributed to the canopy walkway, is retained in a special trust fund managed by the Cape Coast-based Ghana Heritage Conservation Trust (GHCT). This arrangement was established through a special agreement involving the Department of Wildlife, Ministry of Finance, the Cape Coast Castle, and USAID’s Central Region tourism project.

At KCA, GHCT personnel collect their share of gate fees at the visitor centre, which they manage alongside the restaurant and tree platforms where visitors can spend the night. GHCT also operates a bottled water company utilizing the Kakum aquifer. Additionally, GHCT runs a tourism guide-training program in Cape Coast for local guides, who support KCA and conduct tours of Cape Coast and Elmina’s historic sites. These guides supplement Wildlife Department staff during peak visitation periods. Under an agreement with the Department of Wildlife, the guides receive 45% of the entrance fee, with 5% going to the National Tour Guides Association and 50% to the Department of Wildlife, which is then funneled to the Consolidated Fund (USAID 2000).

While the revenue-sharing scheme at KCA has been somewhat effective in maintaining visible facilities like the canopy walkway, visitor centre, and tree platforms, it has not fully ensured efficient day-to-day park management due to the government policy requiring all national park entrance fees to be sent to the consolidated fund, preventing the Wildlife Department from retaining and using its share of the fees directly at KCA or other parks. The inability of the park to compensate farmers means farmers are compelled to resort to lethal methods against wildlife, often resulting in the killing of these animals (Amoah and Wiafe 2012; Binlinla et al. 2014). Although the effectiveness of compensation as a tool for mitigating HWC is debated, it remains widely implemented globally (Ravenelle and Nyhus 2017). To enhance the efficacy of compensation schemes, a prevalent recommendation is to condition payments on the adoption of HWC preventive measures. Incorporating preventive measures into compensation schemes can serve as a proactive approach to mitigate HWC. By requiring farmers to adopt strategies such as building pepper fences, using deterrents, or altering farming practices to prevent wildlife incursions, the likelihood of crop damage can be reduced, and the coexistence between humans and wildlife can be improved. This approach not only addresses the immediate economic losses faced by farmers but also promotes sustainable farming practices and wildlife conservation.

In some African countries, some governments and NGOs manage HWC by using compensation schemes (Bulte and Rondeau 2005, Ogra and Badola 2008, Anthony and Swemmer 2015) whereby payments are made to people in the event of a loss such as human death, injuries, livestock loss, or crop raids. Such schemes aim to increase tolerance levels and prevent retaliatory killings (Muruthi 2005). However, some researchers have argued against it and theorize that paying compensation does not resolve the root causes of HWC (Bulte and Rondeau 2005). Though compensation schemes have been successful in some circumstances (Anthony and Swemmer 2015; Anthony 2021), they are often expensive and bridled with challenges such as bureaucratic inadequacies, corruption, and fraudulent claims (Muruthi 2005). For example, a pilot compensation scheme by Friends of Nairobi National Park aimed at compensating farmers in the event of loss of livestock to predators proved too expensive to continue (Muruthi 2005).

Integrating compensation with preventive measures can create a sense of shared responsibility among stakeholders (Ravenelle and Nyhus 2017). Farmers would be incentivized to engage in conservation-friendly practices, while authorities could ensure that compensation funds are used effectively. This symbiotic relationship can help build trust between communities and conservation agencies, fostering a more collaborative environment for addressing HWC. Implementing such a framework in Ghana could be challenging due to resource constraints and the need for robust monitoring and verification systems. However, learning from successful models elsewhere and adapting them to the local context could provide a viable pathway for mitigating HWC. Ultimately, a well-designed compensation scheme, coupled with preventive measures, could significantly reduce the negative impacts of HWC on both farmers and wildlife, promoting a more harmonious coexistence.

Addressing the intricate relationship between poverty and HWC requires comprehensive strategies that encompass alternative livelihood opportunities, sustainable resource management, and effective conflict mitigation measures. By tackling poverty and empowering communities to adapt to HWC, it becomes feasible to alleviate negative consequences and cultivate resilient and sustainable ecosystems. Providing alternative livelihood opportunities is crucial in reducing dependence on agricultural activities that often lead to HWC. In KCA, implementing alternative livelihoods schemes will not only reduce the pressure on natural resources but also enhance the economic resilience of these communities, making them less vulnerable to the impacts of HWC. By adopting a holistic approach that integrates alternative livelihoods, sustainable resource management, effective conflict mitigation, community empowerment, and supportive policies, it is possible to address the complex interplay between poverty and HWC. This approach can lead to the alleviation of negative impacts, fostering resilient communities and sustainable ecosystems.

### Unequal Exposures and Impacts of HWC

In KCA, we noted that the impact of HWC is not uniform. HWC exacerbates socio-economic disparities by hindering the recovery processes of vulnerable households. This is because disadvantaged populations are unable to put in place effective mitigation measures, thereby increasing their farms’ exposure to crop-raiding by elephants. Thus, within the crop-raiding zones, disadvantaged populations face heightened vulnerability. Their farms frequently suffer severe or total damage due to the lack of adequate deterrent measures. In contrast, economically advantaged farms sustain less damage as these households can afford preventive measures such as pepper fences and additional labour to guard their fields. Additionally, disadvantaged households are more susceptible to HWC because their investments are not diversified. They lack alternative options and are compelled to farm in a single geographical location, even if it is prone to crop-raiding. This situation contrasts sharply with their counterparts whose superior socio-economic status enables them to sustain less impact.

The unequal impacts of HWC are not confined to the KCA. In Kenya’s Amboseli-Tsavo ecosystem, disadvantaged households often face higher incidences of crop-raiding and significant agricultural losses due to their lack of financial resources to implement effective deterrent measures, such as electric fences, leading to exacerbated socio-economic disparities (Kioko et al. 2006). Similarly, in Zimbabwe’s Greater Limpopo Transfrontier Conservation Area, poorer households are disproportionately affected by HWC because they cannot afford protective infrastructure, trapping them in a cycle of poverty (Mhuriro-Mashapa et al. 2018). The situation is mirrored in Tanzania’s Selous-Niassa corridor, where economically disadvantaged families suffer more from crop-raiding incidents, further deepening existing socio-economic inequalities (Kaswamila et al. 2007).

Socio-economic inequality directly translates to fewer resources available for disadvantaged populations to implement effective coping and recovery strategies, thus exacerbating their relative socio-economic conditions. Disadvantaged households and individuals often experience slower recovery from the adverse effects of HWC, which further intensifies existing inequalities. Due to a lack of resources, these populations frequently resort to coping mechanisms that jeopardize their future adaptive capacities and growth potential. For instance, asset-poor households are more likely to provide substandard nutrition to their children and rely on under-resourced public schools, potentially affecting long-term development and prospects. Furthermore, many underprivileged families withdraw their children from school to help guard their farms, compromising their future health and education. Thus, resource scarcity drives them to respond to HWC threats in ways that compromise their future adaptability and growth (Lamarque et al. 2009). Furthermore, disadvantaged households often reduce consumption and investments in human capital to dangerously low levels to preserve their limited physical assets, which has detrimental long-term impacts on the health and education of family members (Ogra 2008; Sitati et al. 2005).

Moreover, underprivileged populations are less equipped to deal with and recover from crop-raiding incidents. For instance, wealthier households can hedge against crop-raiding losses through diversified businesses, a luxury that economically disadvantaged groups cannot afford. Even if both affluent and impoverished families experienced equal exposure and susceptibility to HWC, the rate of recovery could significantly influence future inequality. If disadvantaged groups recover more slowly than advantaged ones, socio-economic disparities will continue to widen.

In all the communities, social capital emerges as a valuable non-tangible asset crucial for coping with the impacts of HWC. Social capital, manifested through social networks, plays a pivotal role in knowledge and information sharing. The better informed a farmer is about effective crop-raiding prevention methods, the stronger their adaptive capacity becomes. In some communities, the significance of social capital, built on trust and reciprocity, is indispensable. Social networks are mobilized to create interventions that mitigate HWC risks. For instance, farmers in communities such as Mfuom and Abrafo form small groups during the farming season to assist each other with planting seeds, weeding, constructing pepper fences, and harvesting, thereby enhancing their ability to deal with crop-raiding. Information exchange and social learning are crucial components of social capital. Social capital facilitates knowledge transfer via social networks. Close relationships with relatives and neighbours form valuable social resources, providing help and encouragement.

### Institutional Influences on the Impact of HWC

In Ghana, the Ministry of Environment, Science, Technology, and Innovation (MESTI) has faced challenges in environmental management and conservation due to financial constraints. Insufficient financial allocation is a significant issue for the ministry. Like many government organizations, MESTI relies on government funding to carry out its operations and programs. However, the allocated budget often falls short of what is needed to address the wide range of environmental activities and challenges the ministry faces, hindering its ability to effectively undertake essential tasks.

Institutional capacity is essential for government agencies like the Wildlife Division, conservation organizations, and other stakeholders to effectively address HWC. However, factors such as funding, staffing, and training can impede the development and implementation of conflict resolution strategies, including those related to HWC. For example, the Wildlife Division office in Abrafo has only three members of staff in the community liaison department responsible for all the communities surrounding the park. Limited financial resources contribute to personnel issues within the ministry, making it challenging to attract and retain skilled experts, limit training opportunities, and impede the hiring of suitable personnel.

Institutional capability significantly impacts the overall outcomes of HWC. If MESTI and its agencies had adequate institutional capacity, they would be capable of developing and implementing policies, engaging stakeholders, monitoring conflicts, providing training and support, enforcing regulations, and fostering collaboration. However, their underfunding means they lack the capacity to deal with HWC, resulting in more frequent and severe confrontations. In response to perceived threats, people resort to unsafe measures to protect their farms and crops. Insufficient community engagement and empowerment also pose challenges, as the Wildlife Division lacks the capacity to actively involve local communities in conflict resolution strategies. A well-funded Wildlife Division could monitor HWC incidents, assess conflict intensity, identify emerging hotspots, and implement warning signals. These constraints increase the vulnerability of communities to HWC incidents, impede the implementation of sustainable mitigation strategies, and prolong conflicts that adversely impact both humans and wildlife. Addressing the financial issues faced by MESTI requires a multifaceted strategy, encompassing increased government funding and improved budget planning and utilization. But this must be driven by shifts in policy priorities.

Policy priorities are areas of utmost importance and urgency that policymakers and government authorities identify, requiring immediate attention and action (Jacoby and Schneider 2001), but are dynamic and can evolve in response to new problems, events, and public sentiment (Castañeda et al. 2018). Policy priorities directly impact the funding and staffing of HWC initiatives. When HWC is prioritized, governments and conservation agencies allocate more resources to mitigation efforts, research, and community support. Adequate resources are crucial for implementing effective measures such as wildlife corridors, early warning systems, and community compensation. Conversely, if HWC is not given sufficient priority, limited resources may result in insufficient or delayed responses, exacerbating conflicts. In countries like Namibia and Bhutan (NPPC and WWF-Bhutan, 2016; MET 2018), HWC policies prioritize proactive approaches, community engagement, and sustainable coexistence between humans and wildlife to address the issue holistically. Since the inception of democratic governance in 1992, HWC has not been a policy priority in Ghana. Instead, policy priorities in Ghana have centred on poverty reduction, healthcare, education, and infrastructure (Awumbila 2006; Whitfield 2005). In many developing countries, poverty reduction is frequently prioritized in development efforts to enhance people’s well-being and livelihoods, addressing basic and urgent needs such as food, housing, clean water, education, and healthcare. These urgent concerns may overshadow the need for comprehensive HWC policies, which are longer-term and indirect in nature. Consequently, long-term environmental issues, including HWC, are overlooked or neglected.

## Conclusion

This study sheds light on the impacts of HWC specifically focusing on crop-raiding activities in KCA. The findings emphasize the challenges posed by elephants in HWC hotspots in rural communities in developing countries. A notable observation is the inability of some residents to fulfil their nutritionary and dietary needs resulting from crop-raiding, jeopardising food security. The study also uncovered the mental health challenges associated with HWC such as fear and anxiety. These stem from the looming threat of harm during encounters with elephants. Furthermore, this study reveals a trend where individuals resort to alcohol as a coping mechanism—a behaviour that is both maladaptive and indicative of the intense psychological distress they experience.

It is crucial for policymakers, conservationists and community stakeholders to collaboratively prioritize strategies that not only tackle the direct challenges associated with crop-raiding but also consider the wider range of social, economic and health related vulnerabilities brought about by HWC. The insights obtained from this study represent a step towards comprehending and effectively managing the broader consequences of HWC. Moreover, although the findings of this research are somewhat unique to the communities in which this research is undertaken, there is potential for them to be applied to other African communities that are facing similar HWC-related challenges. The potential applicability lies in the commonalities of the underlying dynamics and coping mechanisms observed. While each community may have its unique context and specificities, the broader patterns of food insecurity, mental and physical health impacts, and coping strategies identified in the communities surrounding KCA are likely to resonate with other regions grappling with HWC. Therefore, the insights gained from this study could serve as valuable reference points for policymakers, conservationists, and community leaders seeking to address HWC in their respective areas. By recognizing the shared vulnerabilities and responses to HWC, stakeholders can adapt and tailor interventions to suit the local context while drawing on the lessons learned from similar experiences elsewhere in Africa.

## Footnotes

1 A staple food in Ghana primarily made of dried cassava flour.
